# Surgical Antimicrobial Prophylaxis in Patients of Neonatal and Pediatric Age Subjected to Eye Surgery: A RAND/UCLA Appropriateness Method Consensus Study

**DOI:** 10.3390/antibiotics11050561

**Published:** 2022-04-22

**Authors:** Sonia Bianchini, Chiara Morini, Laura Nicoletti, Sara Monaco, Erika Rigotti, Caterina Caminiti, Giorgio Conti, Maia De Luca, Daniele Donà, Giuseppe Maglietta, Laura Lancella, Andrea Lo Vecchio, Giorgio Marchini, Carlo Pietrasanta, Nicola Principi, Alessandro Simonini, Elisabetta Venturini, Rosa Longo, Elena Gusson, Domenico Boccuzzi, Luca Vigo, Fabio Mosca, Annamaria Staiano, Susanna Esposito, on Behalf of the Peri-Operative Prophylaxis in Neonatal and Paediatric Age (POP-NeoPed) Study Group

**Affiliations:** 1 Pediatric Clinic, University Hospital, Department of Medicine and Surgery, University of Parma, 43126 Parma, Italy; bianchini.sonia@outlook.it (S.B.); laura.nicoletti@studenti.unipr.it (L.N.); s.monaco1410@gmail.com (S.M.); 2 Ophthalmology Unit, Guglielmo da Saliceto Hospital, 29121 Piacenza, Italy; c.morini@ausl.pc.it; 3 Pediatric Clinic, Azienda Ospedaliera Universitaria Integrata, 37134 Verona, Italy; erika.rigotti@aovr.veneto.it; 4 Clinical and Epidemiological Research Unit, University Hospital of Parma, 43126 Parma, Italy; ccaminiti@ao.pr.it (C.C.); gmaglietta@ao.pr.it (G.M.); 5 Pediatric ICU and Trauma Center, Fondazione Policlinico Universitario A. Gemelli IRCCS, 00165 Rome, Italy; giorgio.conti@unicatt.it; 6 Paediatric and Infectious Disease Unit, Academic Department of Pediatrics, IRCCS Bambino Gesù Children’s Hospital, 00165 Rome, Italy; maia.deluca@opbg.net (M.D.L.); laura.lancella@opbg.net (L.L.); 7 Division of Paediatric Infectious Diseases, Department for Woman and Child Health, University of Padua, 35100 Padua, Italy; daniele.dona@unipd.it; 8 Department of Translational Medical Science, Section of Pediatrics, University of Naples “Federico II”, 80138 Naples, Italy; andrealovecchio@gmail.com (A.L.V.); fabio.mosca@unimi.it (F.M.); staiano@unina.it (A.S.); 9 Ophthalmology Unit, Azienda Ospedaliera Universitaria Integrata, 37134 Verona, Italy; giorgio.marchini@univr.it (G.M.); rosa.longo@univr.ir (R.L.); elena.gusson@aovr.veneto.it (E.G.); 10 NICU, Fondazione IRCCS Ca’ Granda Ospedale Maggiore Policlinico, Department of Mother, Child and Infant, 20122 Milan, Italy; carlo.pietrasanta@unimi.it; 11 Università degli Studi di Milano, 20122 Milan, Italy; nicola.principi@unimi.it; 12 Pediatric Anesthesia and Intensive Care Unit, Salesi Children’s Hospital, 60123 Ancona, Italy; dr.simonini@gmail.com; 13 Pediatric Infectious Diseases Unit, Meyer Children’s Hospital, 50139 Florence, Italy; elisabetta.venturini@meyer.it; 14 Carones Vision Advalia, 20145 Milan, Italy; dboccuzzi@carones.com (D.B.); lvigo@carones.com (L.V.)

**Keywords:** antibiotics, eye, pediatric infectious diseases, pediatric ophthalmology, ocular surgery, surgical antimicrobial prophylaxis

## Abstract

Ocular surgery encompasses a wide range of procedures, including surgery of the tear ducts, eyelid, cornea and conjunctiva, lens, ocular muscle, and vitreoretinal and iris surgery. Operations are also performed for the removal of tumors, repairs of ocular trauma and, finally, corneal transplantation. Antibiotic prophylaxis for the prevention of surgical site infections (SSIs) in ocular surgery is a complex field in which shared lines of action are absent. In light of the scarcity of shared evidence in the use of ocular antimicrobial prophylaxis for the pediatric population, this consensus document aims to provide clinicians with a series of recommendations on antimicrobial prophylaxis for patients of neonatal and pediatric age undergoing eye surgery. The following scenarios are considered: (1) intraocular surgery; (2) extraocular surgery; (3) ocular trauma; (4) ocular neoplasm; (5) ocular surface transplantations; (6) corneal grafts. This work has been made possible by the multidisciplinary contribution of experts belonging to the most important Italian scientific societies and represents, in our opinion, the most complete and up-to-date collection of recommendations regarding clinical actions in the peri-operative environment in eye surgery. The application of uniform and shared protocols aims to improve surgical practice, through the standardization of procedures, with a consequent reduction of SSIs, also limiting the phenomenon of antimicrobial resistance.

## 1. Introduction

Ocular surgery encompasses a wide range of procedures, including surgery of the tear ducts, eyelid, cornea and conjunctiva, lens, ocular muscle, and vitreoretinal and iris surgery. Operations are also performed for the removal of tumors, repairs of ocular trauma and, finally, corneal transplantation [1]. Table 1 summarizes major ocular surgeries in patients of neonatal and pediatric age.

Antibiotic prophylaxis for the prevention of surgical site infections (SSIs) in ocular surgery is a complex field in which shared lines of action are absent. Since many articles and reviews focus on the adult population, this absence burdens the pediatric population even more. In light of the scarcity of shared evidence in the use of ocular antimicrobial prophylaxis for the pediatric population, this consensus document aims to provide clinicians with a series of recommendations on antimicrobial prophylaxis for patients of neonatal and pediatric age undergoing eye surgery.

## 2. Methods

### 2.1. RAND/UCLA Method of Appropriateness

This paper was created with the application of the RAND/UCLA (Research and Development Corporation of the University of California—Los Angeles) method of appropriateness, which consists of expert evaluation of the appropriateness of diagnostic, managerial, and therapeutic procedures with suboptimal scientific evidence [2]. According to the RAND method, a procedure is defined as “appropriate” when the expected benefits outweigh the expected adverse consequences, whereas a procedure where expected risks exceed the expected benefits is defined as inappropriate. According to the RAND definition, experts must make a judgment of appropriateness/inappropriateness by considering only the clinical benefits, without making economic considerations [3]. For a heterogeneous topic such as surgical antimicrobial prophylaxis on which randomized controlled trials in pediatrics are lacking, the application of methods aiming to increase the homogeneity of clinical actions by neonatologists, infectious diseases specialists, pediatric surgeons, and anesthetists appeared useful and appropriate. For this reason, the RAND/UCLA approach was chosen instead of GRADE methodology. Through the RAND method, the participants discussed different clinical scenarios and elaborated statements based on the published literature and their clinical experience. The group of experts did not consider it appropriate to combine the GRADE method with the RAND/UCLA approach because the absence of randomized studies represents a bias in defining the strength of the recommendations and in representing a consensus reached for real-life.

### 2.2. Recruiting the Expert Panel

A multidisciplinary group of experts belonging to the main Italian scientific societies was selected, composed of pediatricians, neonatologists, specialists in infectious diseases, pediatric surgeons, anesthesiologists, pharmacologists, microbiologists, and ophthalmologists. The following Scientific Societies were involved: the Italian Society of Pediatrics (SIP), the Italian Society of Neonatology (SIN), the Italian Society of Pediatric Infectious Diseases (SITIP), the Italian Society of Infectious and Tropical Diseases (SIMIT), the Italian Society of Pediatric Surgery (SICP), the Italian Society of Microbiology (SIM), the Italian Society of Pharmacology (SIF), the Italian Society of Neonatal and Pediatric Anesthesia and Resuscitation (SARNEPI), and the Italian Society of Childhood Respiratory Diseases (SIMRI). The panel of experts was made up of 52 medical doctors with at least 5 years of experience: pediatricians (*n* = 20), neonatologists (*n* = 6), infectious diseases specialists (*n* = 5), pediatric surgeons and ophthalmologists (*n* = 5), anesthetists (*n* = 8), pharmacologists (*n* = 5), and microbiologists (*n* = 3).

### 2.3. Scenario Formulation

A literature search was performed with a selection of papers including randomized trials, systematic literature reviews, meta-analyses, and guidelines on peri-operative prophylaxis for the prevention of SSIs in eye surgery. The literature search was performed on the PubMed database, with a selection of English-language articles published from 2000 to 2020. In cases in which the literature of the above years was scarce, the search was expanded to include work from 1980 onward. The key search terms were “antimicrobial prophylaxis” OR “antibiotic prophylaxis” AND “oculistic” OR “cataract” OR “glaucoma” OR “dacryocystitis” OR “intracameral antibiotic” OR “strabismus” OR “blepharoplasty” OR “ocular trauma” OR “foreign body” OR “ocular/eye” OR “retinopathy of prematurity” OR “transplant/transplantation” OR “ocular neoplasia” OR “ocular tumor” OR “corneal grafts” AND “neonate” OR “newborn” OR “paediatric” OR “pediatric” OR “children” OR “adolescent”.

Next, using the Patient/Problem/Population-Intervention-Comparison/Control/Comparator-Outcome (PICO) model, a questionnaire on peri-operative antimicrobial prophylaxis in eye surgery was created for the pediatric and neonatal population, divided into six clinical scenarios. Before administration, it was tested twice with a one-week interval to a convenience sample of four pediatricians, two neonatologists, one infectious diseases specialist, one ophthalmologist, one anesthetist, one pharmacologist and one microbiologist. Then, 26 out of 52 experts were selected by the scientific societies for answering and the questionnaire was administered to 11 pediatricians, 3 neonatologists, 2 infectious diseases specialists, 3 ophthalmologists, 4 anesthetists, 2 pharmacologists, and one microbiologist.

### 2.4. Two-Round Consensus Process

Based on the scenarios, the questionnaire was submitted to experts on the online platform “REDCap”. REDCap (Research Electronic Data Capture) is a secure, web-based software platform designed to support data capture for research studies, providing: (1) an intuitive interface for validated data capture; (2) audit trails for tracking data manipulation and export procedures; (3) automated export procedures for seamless data downloads to common statistical packages; and (4) procedures for data integration and interoperability with external sources. The selected literature was made available to all panel members, who were instructed on how to complete the questionnaire. Experts answered the questionnaire anonymously and their rating was expressed on a scale from 1 (definitely inappropriate) to 9 (definitely appropriate). The intermediate values correspond to different modulations of the judgment of inappropriateness (“2” and “3”), uncertainty (from “4” to “6”), and appropriateness (“7” and “8”). In evaluating each indication, experts relied on available scientific evidence and their own clinical experience and judgment. Free space was, in addition, provided for any annotations or comments.

The first round of the questionnaire was blinded to the other panel members. Multiple participation was not permitted by the platform, which also guaranteed the confidentiality and anonymity of the answers. The results of the survey were discussed in a collegial meeting with all the 26 experts who answered the questionnaire to reach agreements and reduce eventual disagreements [3]. Clarifications, adaptations, and refinements of the claims and adequacy ratings were made. A total of six recommendations were developed. Participants were asked to endorse the recommendations in a second round during the following 4 weeks.

## 3. Results

### 3.1. SCENARIO #1. Pediatric Patient Undergoing Intraocular Surgery

Intraocular surgery includes surgery of the lens, trabecular meshwork, cornea if a full-thickness cut is performed, iris and vitreoretinal surgery. In pediatric age, the most frequent intervention among these is the extraction of cataracts. Vitreoretinal surgery concerns in particular the intervention on retinopathy of the premature. No prospective studies are available regarding antimicrobial prophylaxis to be administered in pediatric patients who are to undergo this type of surgery.

Intraocular surgery can be complicated by acute endophthalmitis, a severe intraocular inflammation due to infection, which usually appears within a week of surgery and can lead to severe outcomes, including vision loss [1]. The predominantly involved microorganisms are Gram-positive bacteria, with a prevalence of coagulase-negative *Staphylococci*, followed by *Staphylococcus aureus*, *Streptococcus* spp. and *Enterococcus* spp.. Gram-negative bacteria involved include *Pseudomonas* spp., *Proteus* spp. and *Haemophilus influenzae* [4].

Therefore, the use of antimicrobial prophylaxis in ophthalmic surgery is, as its main objective, to reduce the occurrence of endophthalmitis. Although the available data regarding the efficacy of antimicrobial prophylaxis in ophthalmic procedures in the adult population are limited, even more in the pediatric population, the administration of antibiotics by the intracameral route is common in clinical practice [1]. There is, however, wide variability regarding the antibiotic agents used, the routes (topical, intraocular, subconjunctival, and oral), and the timing of administration (preoperative, intraoperative, peri-operative, and postoperative) [5].

The review by Rahmani and colleagues on postoperative endophthalmitis in intraocular surgery procedures for cataract, glaucoma, and vitrectomy surgery first emphasizes the critical role of povidone iodine in prevention and, based on recent studies, also suggests the use of prophylactic intracameral antibiotics for further reduction in the rate of endophthalmitis. The most commonly used molecules are cefuroxime, moxifloxacin, and, less frequently, vancomycin. The use of intracameral antibiotics is not without side effects, so the authors conclude by stating that their mandated use cannot be indicated [4].

Bratzler and colleagues, in guidelines developed by the American Society of Health-System Pharmacists, the Infectious Diseases Society of America, the Surgical Infection Society, and the Society for Healthcare Epidemiology of America, also state that, because of the lack of support from validated studies, specific recommendations cannot be made regarding antimicrobial prophylaxis in ocular surgery. Appropriate topical antimicrobials include a commercially available neomycin-polymyxin B-gramicidin solution or fluoroquinolones administered as one drop every 5 to 15 min for five doses within an hour before the start of the procedure. The addition of subconjunctival cefazolin 100 mg, intracameral cefazolin 1–2.5 mg, or cefuroxime 1 mg at the end of the procedure is optional [1].

Huang and colleagues, in an extensive systematic literature review and meta-analysis, investigated the use and route of administration of antimicrobials in the prevention of postoperative endophthalmitis. The authors conclude by stating that intracameral antibiotics are effective in preventing endophthalmitis. The molecules used are cefuroxime, whose efficacy is confirmed by the data analyzed in the paper, while other studies support the efficacy of vancomycin/moxifloxacin. Regarding topical therapy, the authors reported discordant clinical data, concluding that there is insufficient evidence to indicate that its use prevents endophthalmitis [6].

Regarding cataract removal surgery specifically, the literature first recommends the use of topical preoperative povidone iodine, which decreases conjunctival bacterial colonization, allowing the incidence of endophthalmitis after cataract surgery to be significantly reduced [7,8,9,10]. Regarding antibiotic use, currently the safest and most effective antibiotic prophylaxis in cataract surgery appears to be the use of intracameral antibiotic at the end of the procedure [5,11,12,13,14,15]. Indeed, the intracameral route provides a much higher concentration of drug at the surgical site, resulting in greater bactericidal activity than the topical route [16,17]. Intracameral injection of antibiotics at the end of the procedure is intended to kill microorganisms that are introduced during surgery through the corneal incisions. In fact, despite the preoperative use of antibiotic eye drops and povidone iodine, careful sterilization, and the application of aseptic protocols, the intraocular bacterial contamination rate remains high, reaching 31% [18]. The most used drugs in intracameral prophylaxis are cephalosporins (cefuroxime or cefazolin), moxifloxacin, or vancomycin. Doses of 1 mg of cefuroxime, 0.1 mg of vancomycin, and 0.5 mg of moxifloxacin have been reported at the end of the procedure [19,20].

The use of topical antibiotics is frequent in clinical practice, both during the preparation for surgery and in the postoperative period, although their use is controversial due to the scarcity of large randomized controlled trials that could confirm the efficacy of their use [21,22]. A large prospective, randomized, controlled trial conducted by the European Society of Cataract and Refractive Surgery (ESCRS) compared the rate of postoperative endophthalmitis in more than 16,000 patients undergoing routine cataract correction procedures at 24 European centers. All patients were randomized and divided into 4 groups on the basis of peri-operative strategies: intracameral administration of cefuroxime at the end of the procedure; application of peri-operative levofloxacin 0.5% ophthalmic solution; combination of intracameral cefuroxime and levofloxacin 0.5% ophthalmic solution; or no antimicrobial prophylaxis. In all patients, disinfection of the ocular area with 5% povidone iodine was performed before surgery and topical levofloxacin was administered in the postoperative period [19,23]. The results reported a significant reduction in endophthalmitis rates in patients who had received intracameral cefuroxime. However, this study has been questioned, given the high rate of endophthalmitis in patients who were not randomized to cefuroxime administration, the lack of an approved commercial preparation, and also because of the risks of hypersensitivity, dilution errors, and contamination inherent in the use of intra-chamber drugs [24,25,26].

A 2017 Cochrane review concluded that the combination of intracameral antibiotic injection and antibiotic eye drops administration can effectively reduce the occurrence of endophthalmitis compared with the two procedures (intracameral injection and eye drops instillation) used individually [27]. In contrast, the American Academy of Ophthalmology Preferred Practice Pattern Cataract, in guidelines published in 2021, reports that intracameral administration of antibiotics, in combination with the use of povidone iodine and an aseptic surgical technique, is sufficient for the prevention of postoperative endophthalmitis, deeming the use of antibiotic eye drops unnecessary [28,29].

An observational study of more than 300,000 procedures in the adult population compared the intracameral administration of cefuroxime or moxifloxacin with the topical use of antibiotic eye drops and concluded that there was a lower risk of infectious complications in patients who received the intracameral antibiotic, whether or not associated with topical therapy. The authors also pointed out that topical therapy alone was less effective, more prone to prescription errors and non-adherence, and specifically advised against the use of topical aminoglycosides because they were ineffective in preventing endophthalmitis [21].

A 2021 online survey involving more than 1200 members of the American Society of Cataract and Refractive Surgery found increasing use of intracameral antibiotics, significantly greater than similar surveys conducted in 2014 and 2007 [30].

Retinopathy of Prematurity (ROP) is a multifactorial vasoproliferative retinal disorder affecting the retina of premature infants, with incidence inversely proportional to gestational age. The International Classification of Retinopathy of Prematurity (ICROP) classification system for ROP is based on location, staging (stage 1 to stage 5), extent, and presence of plus disease. The treatment is based on laser photocoagulation, cryotherapy, therapy with anti-endothelial growth factor drugs (anti-VEGF9 drugs), or surgery. The latter belongs to the group of intraocular surgery and is applied when retinal detachment has occurred (i.e., in the most severe stages, stages 4 and 5). In these occasions the surgical approach involves a vitrectomy, in an attempt to eliminate the traction forces on the retina and reattach it [31,32,33]. However, there are no specific indications in the literature regarding the eventual antibiotic prophylaxis to be administered in ROP surgery.

**Recommendation 1.** In the pediatric patient undergoing clean intraocular eye surgery, peri-operative prophylaxis with 5% povidone iodine instilled on the ocular surface 3–5 min before surgery, 10% povidone iodine for disinfection of the peri-ocular skin, and intracameral cefuroxime at a dosage of 1 mg at the end of the procedure are recommended. No specific systemic antibiotic prophylaxis is recommended.

### 3.2. SCENARIO #2. Pediatric Patient Undergoing Extraocular Surgery

Extraocular surgeries are considered clean surgeries and include procedures involving the eyelids, lacrimal glands, muscles of the eye, conjunctiva, and cornea, when the entire thickness is not affected. Of these, the one most frequently performed in the pediatric population is correction of strabismus. No prospective studies are available regarding the antimicrobial prophylaxis to be administered in pediatric patients undergoing this type of surgery. There is, however, uniformity of thought regarding the importance of using povidone iodine, in combination with an aseptic procedure, to prevent SSIs. The microorganisms most frequently involved are bacteria belonging to the ocular and conjunctival flora, with variations based on the age of the patients. In particular, in children *Streptococcus* spp. are isolated more frequently [34].

With regard to blepharoplasty surgery, Ferneini and colleagues, in an extensive and recent review of the literature on the use of antibiotic prophylaxis in this procedure in the adult population, concluded that the currently available data cannot direct an unambiguous course of action. Therefore, although the overall rate of infection associated with eyelid surgery is extremely low, there is no standard of care to adhere to with regard to peri-operative antimicrobial prophylaxis [35].

Regarding antimicrobial prophylaxis in tear duct surgery, the use of systemic antibiotics in tear drainage surgery is controversial [36]. Sheth and colleagues, in a prospective randomized controlled trial, compared the efficacy of a single peri-operative dose of intravenous antibiotic versus postoperative oral antibiotic prophylaxis for the prevention of SSIs in external dacryocystorhinostomy in the adult population. Patients in the first group received a single bolus dose of intravenous cefazolin 1 g during surgery, whereas those in the second group received oral cephalexin 500 mg after surgery twice daily for 5 days. The results demonstrated that the efficacy in SSI prevention of a single dose of peri-operative intravenous antibiotic was comparable to that offered by the most widely used oral antibiotic prophylaxis [37].

In contrast, Pinar-Sueiro and colleagues, in a study conducted in the adult population regarding the prophylactic use of antibiotics in external dacryocystorhinostomy, observed that the use of antibiotics during surgery was not associated with a lower rate of postoperative infectious complications. However, a statistically significant association was found between some specific clinical pictures and a higher rate of positive cultures. Based on these data, the authors question the widespread use of prophylactic antibiotics for external dacryocystorhinostomy, recommending, however, their use in patients with previous episodes of mucocele, mucopiocele, or acute dacryocystitis [38]. Similarly, Dulku and colleagues, who conducted a retrospective study evaluating the rate of postoperative infections after external dacryocystorhinostomy without antibiotic prophylaxis, concluded that, considering the rarity of infectious consequences in this type of surgery, the routine use of systemic antibiotic prophylaxis may not be warranted [39].

Postoperative infections in strabismus surgery may present as endophthalmitis, subconjunctival abscess, subtenonic abscess, orbital cellulitis, or orbital abscess [40]. The risk of endophthalmitis in strabismus surgery represents a rare occurrence, with an incidence that is difficult to determine and estimated to be between one case in every 35,000 to one in 185,000, with severe visual outcomes [41]. In strabismus surgery, the causes of endophthalmitis can be multiple and are reported even without evidence of ocular globe perforation. In these cases, the infection could spread through endogenous dissemination, extension of a scleral abscess, extension of an infection involving the suture at the site of muscle reinsertion, or bacteria deposited by the suture along its intra-scleral pathway.

In strabismus surgery, the current standard of care involves cleansing and disinfection of the eyelids, eyelashes, and peri-ocular area with 10% povidone iodine and application of 5% povidone iodine drops into the eye during surgical preparation, with evidence of decreased incidence of postoperative infections [40]. Some works specifically address the application of the antiseptic iodine povidone, the importance of which remains paramount in strabismus surgery. The study by Benson and colleagues investigated the rate of contamination (defined as any bacterial growth on 2-day blood agar plates) of the surgical site in patients who received a single application of 5% povidone iodine, compared with a double application. The results of the study showed a significant reduction in the rate of surgical site contamination with double administration [42].

In a comparative study, Koederitz and colleagues compared the use in the postoperative period of single-dose 5% povidone iodine drops versus a one-week course of antibiotics and steroids as prophylaxis against postoperative infection in extraocular muscle surgery. The authors conclude that single-dose povidone iodine is a cost-effective alternative to postoperative antibiotic/steroid prophylaxis after routine strabismus surgery performed through a fornix incision. Postoperative antibiotic/steroid use might, however, be recommended in patients who have undergone reinterventions and limbal incisions [43].

A recent multicenter study by DeBenedictis et al. investigated practice patterns for infection prophylaxis in pediatric ophthalmic strabismus surgery. Specialists (members of the American Association for Pediatric Ophthalmology and Strabismus) responded to a survey (completed by 380 members) that showed 88.4% use topical administration of 5% povidone iodine during surgical preparation, 90% use topical antibiotics with or without steroids at the end of surgery, and 85.5% prescribe oral or topical antibiotics after surgery [44].

**Recommendation 2.** In the pediatric patient undergoing clean extraocular eye surgery, peri-operative prophylaxis with 5% povidone iodine instilled on the ocular surface 3–5 min before surgery and 10% povidone iodine for disinfection of the peri-ocular skin are recommended. Routine specific topical or systemic antibiotic peri-operative prophylaxis is not recommended, except in subjects with recurrence of infection at the site involved by the surgery.

### 3.3. SCENARIO #3. Pediatric Patient with Ocular Trauma

Ocular trauma is a major cause of disability in all ages, but its impact is particularly negative in the pediatric population [45]. It represents the leading cause of monocular blindness in children, despite the fact that approximately 90% of ocular traumas are preventable [46,47,48,49]. Thompson et al. reported that approximately 20–50% of hospital admissions for ocular trauma occur in pediatric subjects [50]. Children with ocular trauma may present with a much more complicated course than adults with comparable trauma (particularly with development of amblyopia). This is compounded by delays in access to care, unclear mechanisms of injury, and difficulty in cooperating with ocular examination [51,52,53].

The following have been reported as common causes of ocular trauma in the pediatric population: penetration and blunt trauma from sticks and toys, bullets, fireworks, and various objects during traffic accidents [45,54,55,56]. The most frequent causes of reduced visual acuity after trauma are amblyopia and corneal opacities. Risk factors for worse prognosis include young age, poor initial visual acuity, posterior involvement, extensive injury, vitreous hemorrhage, retinal detachment, and endophthalmitis [57]. Ram et al. reported a large case series of ocular trauma (distinguishing between penetrating and non-penetrating) in subjects aged 1 to 15 years, reporting significant visual impairment in trauma-affected eyes resulting in traumatic cataracts [58]. Yardley’s group reported, in a comprehensive review, the different types of animals involved in determining ocular or adnexal trauma, which can be potentially devastating from both an aesthetic and functional point of view [59]. Although with differences arising from case histories and geographic location, ocular trauma caused by insects, primarily ophthalmomyasis, is the most frequently reported [60]. Less than 5% of ocular trauma is animal-related. Dogs represent the animals most often involved in adnexal damage, especially at the canalicular level, whereas birds and non-domestic mammals are responsible for the most severe trauma [61].

Several score systems have been proposed to predict visual acuity, in particular the Ocular Trauma Score (OTS) and the Pediatric Ocular Trauma Score (POTS), with the aim of improving communication between emergency room personnel and the ophthalmologist and to be able to determine a more accurate prognosis [57,62,63]. While Awidi’s group advocates the use of the POTS score, Pahor and colleagues, in a more recent review of 36 cases (subjects <19 years of age), believe that the OTS is more accurate in defining the probability of visual impairment and is more readily used [51,64]. Most of the research related to ocular trauma focuses on epidemiological characterization, in terms of incidence, population most affected, and type and modality of trauma, without focusing on the subsequent intervention and the use (or not) of antibiotic prophylaxis [45]. Upadhyay et al. reported in a large case series of patients, in a low-resource setting, that the use of a topical 1% chloramphenicol ointment administered as prophylaxis within 18 h of ocular trauma, with evidence of corneal abrasion, had very high efficacy in preventing the development of corneal ulceration [65]. Regarding the use of antimicrobial prophylaxis in penetrating trauma, an experimental study in the animal model conducted by Alfaro’s group reported excellent intravitreal penetration of cefazolin, with levels above the minimal inhibitory concentration for most microorganisms [66]. In the specific case of ocular and adnexal trauma due to dog bite, Erickson’s group reported an oral antibiotic prophylaxis (with azithromycin) to be useful, especially in subjects with important involvement of the structures and who present late to treatment; however, these patients were not then submitted to surgery [67].

Lou et al. reported the outcome of a large survey administered to ophthalmologists evaluating the use of antibiotic prophylaxis in open eye trauma. In particular, 56.9% of respondents reported administering antibiotics by intraocular injections (intracameral or intra-vitreal) only when there was a high risk of endophthalmitis (lens capsule rupture, persistence of foreign body in place, delayed wound closure, dirty wound, large wound, inflammatory reaction before wound repair). The predominantly administered molecules were cephalosporins, followed by vancomycin [68]. Pouzaud’s group emphasized, in an in vitro study, the good ocular diffusion of ofloxacin and levofloxacin, their broad spectrum of action, and showed reduced tissue toxicity [69]. Studies demonstrated a reduction in the risk of endophthalmitis in subjects undergoing antibiotic prophylaxis after ocular repair for trauma, particularly with intravenous administration of vancomycin and ceftazidime or with vancomycin and cefepime [70,71,72]. In contrast, Abouammoh’s group reported a reduction in the risk of endophthalmitis with intravitreal injection of vancomycin and ceftazidime alone, at the end of the first trauma repair, with the caution of injecting the combination of antibiotics gradually to avoid increasing intraocular pressure and thus leading to a rupture of the recent repair [73].

**Recommendation 3.** In the pediatric patient undergoing surgery for penetrating trauma of the eye, 10% povidone iodine for disinfection of the peri-ocular skin and intraocular antibiotic prophylaxis with vancomycin and ceftazidime are recommended. It is also recommended to administer vancomycin 15 mg/kg (max 1 g) IV in combination with ceftazidime 50 mg/kg (max 2 g) IV in cases in which the risk of infection is increased (i.e, age <1 year, poor initial visual acuity, posterior involvement, extensive injury, vitreous hemorrhage, retinal detachment, and endophthalmitis).

### 3.4. SCENARIO #4. Pediatric Patient Undergoing Ocular Surgery for Neoplasm

All ocular structures can be affected by tumors, but these tumors can occur at different stages of life [74]. Table 2 summarizes the main neoplasms with therapeutic approaches, divided into surgical vs non-surgical. Intraocular lymphoma, a rare entity, can primarily involve several structures: retina, vitreous, ciliary body, choroid. Metastases tend to involve the uvea more frequently and may be due to various systemic neoplasms.

Retinoblastoma is the most common malignant ocular tumor in the pediatric population, predominantly in younger subjects, with most cases diagnosed before 2 years of age and 80% by 4 years of age. An incidence of 1 per 18,000 new births is reported [74,75,76,77]. It is suspected in the presence of leukocoria or photoleukocoria, or strabismus. The differential diagnosis is with persistent fetal vascularization, Coats’ pathology, vitreous hemorrhage, retinopathy of the premature, coloboma and congenital cataract [78,79,80,81,82]. If left untreated, it has a high lethality and a high risk of visual impairment [75]. Therefore, therapy must be started early and be individualized, based on the severity of the pathology, laterality (mono or bilateral), age of the patient, resorting to different therapeutic options: focal conservative modalities (laser hyperthermia/photocoagulation and cryotherapy), surgical enucleation, chemotherapy and possibly radiotherapy in refractory cases [83]. Chemotherapy often represents a “bridging therapy” in subjects younger than 3–6 months or less than 7 kg in weight who cannot undergo options such as intra-artery chemotherapy/ophthalmic artery surgery, keeping in mind the increased risk of chemotherapy side effects (ototoxicity and peripheral neuropathy) in this age group [84,85,86,87]. Lin’s group reports that no randomized controlled trials on retinoblastoma therapy are available in the literature and points out that this type of study is difficult to perform [75].

Conjunctival nevi account for approximately 61% of pediatric cancers, with onset predominantly in the second half of the first decade of life [88,89]. In the case series of 59 subjects followed by Negretti and colleagues, progression was reported in 22% of cases [90]. No studies are available in the literature that focus on whether or not specific peri-operative antibiotic prophylaxis is used in this type of subject.

**Recommendation 4.** In neonatal and pediatric subjects undergoing ocular surgery for removal of neoplasm (minimally invasive surgery, laser therapy, enucleation), 10% povidone iodine for disinfection of the peri-ocular skin is recommended. On the contrary, the routine use of peri-operative prophylaxis is not recommended.

### 3.5. SCENARIO #5. Pediatric Patient Undergoing Ocular Surface Transplantations

Most of the work in the literature focuses on ocular issues related to other organ transplantation in pediatric patients [91]. The Fernández Jiménez-Ortiz Group reviewed data on amniotic membrane transplantation in cases of reduced ocular motility, both as a therapy and as a preventive measure in cases of restrictive strabismus from muscle adhesion. The review reported a favorable outcome of the procedure in subjects with a mean age of 21 years, but did not go into detail about whether or not to adopt peri-operative antibiotic prophylaxis [92]. The work of Iyer and colleagues reported the successful experimental therapy of an allo-Simple Limbal Epithelial Transplantation (allo-SLET) in 3 adult subjects after surgical removal of superficial squamous neoplasia, with postoperative use of 0.04% mitomycin C, 2 cycles, every other week [93]. Patil’s group reported good success in the combination of amniotic membrane transplantation and 0.02% mitomycin applied to resected papillae in 4 pediatric patients (7–14 years old) treated for vernal keratoconjunctivitis, but did not mention pre- and intra-operative antibiotic therapy [94].

**Recommendation 5.** In pediatric subjects undergoing ocular surgery for amniotic membrane or limbal transplantation it is recommended to complete an evaluation with a multi-specialist team to define the correct peri-operative prophylaxis.

### 3.6. SCENARIO #6. Pediatric Patient Undergoing Corneal Grafts

Corneal grafts (keratoplasty) have progressed enormously in the last two decades due to the development of new surgical techniques. The graft of the whole cornea (penetrating keratoplasty) has been progressively replaced by lamellar keratoplasties, in which specific tissues of the cornea are grafted (epithelium/stroma, endothelium, or Descemet/endothelium) in various conditions. This has led to a dramatic reduction of rejection rates as well as other post-surgical complications, and ultimately to improved visual outcomes [95].

Despite this enormous progress, corneal grafts in the pediatric population present with totally different challenges when compared to the adult population, and remain regarded as a high-risk procedure offered to few groups of patients with specific indications, such as corneal opacities in congenital glaucoma, anterior segment dysgenesis, and post-infectious scars [96,97]. Unique challenges are rejection risks (occurring at a higher percentage when compared to adults), demanding postoperative management (often requires long-term instillation of eye drops, frequent consultations, multiple evaluations under narcosis), and infection rates. Some authors advocate prophylaxis with long-term postoperative use of topical antibiotics (polymyxim B-trimetoprim and quinolones) for many months until sutures are removed [98,99,100]. When infection of the graft occurs, an aggressive management is recommended with topical as well as systemic antibiotics. Evidence-based data to guide the choice of antibiotic class and dosage are not available due to the rarity of the surgical applications.

**Recommendation 6.** In pediatric subjects undergoing corneal grafts (keratoplasty) it is recommended to complete an evaluation with a multi-specialist team to define the correct peri-operative prophylaxis.

## 4. Discussion

Endophthalmitis after ophthalmic surgery is, fortunately, a rare occurrence. However, since the visual prognosis is generally unfavorable and the complication multifactorial, all efforts should be made to minimize the risk of endophthalmitis. The data available in the literature do not allow an unambiguous interpretation, both because they largely concern the adult population, burdened by various risk factors, and because of the scarcity of data and prospective studies. What emerges is that, among the intraocular ophthalmic surgery, cataract surgery is the one on which studies on peri-operative antimicrobial prophylaxis have focused, with discordant results regarding the choice of molecules, but substantial agreement on the opportunity to perform prophylaxis with antibiotics administered intracamerally. Optimization of surgical conditions to reduce complications and duration of surgery and the use of subconjunctival and intracameral antibiotics with activity against Gram-positive microorganisms may reduce the incidence of endophthalmitis following pediatric cataract surgery. Finally, it should be emphasized that the choice of the type of intracameral antibiotic in the different case series depends on the experience of the surgeon, the clinical setting, and the availability of the drug as an approved and marketed formulation. In fact, frequently the non-use of intracameral antibiotics depends mainly on the non-availability of antibiotic preparations approved for intracameral use [101]. The panel, based on literature data and personal experience, recommend, in agreement with the literature, the use of intracameral antibiotics as prophylaxis in intraocular surgery, but do not recommend the use of systemic antibiotics. Intracameral administration of antibiotics is considered off-label by most drug regulatory agencies. Interestingly, in the UK, cefuroxime (i.e., the most extensively studied antibiotic for its efficacy and safety in intracameral injection) has been approved for use in intracameral injections [102]. It is important to highlight that isotonic preparation should be with 0.9% NaCl and free of preservatives or other additives.

Regarding ROP surgery, in the absence of shared guidelines regarding antimicrobial prophylaxis, the expert panel has opted to standardize the indication for this specific procedure to that of intraocular surgery overall.

With regard to extraocular surgery, no prospective, randomized studies are available regarding the use of antimicrobial prophylaxis in this type of procedure in the pediatric population. However, there is no unequivocal recommendation in the adult population, and peri-operative strategies depend primarily on the patient’s underlying conditions, the presence of pre-existing infections, and the experience of the ophthalmologist. Considering the low incidence of SSIs reported in this type of surgery, the expert panel concludes at not recommending the administration of peri-operative antibiotics either intracamerally or systemically, except in cases where a particular recurrence of infection is reported at the site of surgery or for specific problems of the patient themself.

With regard to trauma-related surgery, the panel recommends the use of intraocular antibiotic prophylaxis, in agreement with the limited available literature [103,104,105]. In view, however, of the increased contamination possible in some circumstances when trauma occurs, the panel emphasizes the possibility of adding intravenous prophylaxis as well.

The literature does not report the use of peri-operative antibiotic prophylaxis in subjects undergoing surgery for removal of ocular neoplasia. The expert panel, based on several factors, such as sterility of the ocular structure, type of surgery using laser or minimally invasive surgery, concludes at not routinely recommending systemic peri-operative antibiotic prophylaxis in this type of surgery.

On the other hand, as far as surgery in case of various forms of ocular transplantation is concerned, the type of intervention is so particular that it cannot be included in the surgical routine and for this reason the panel of experts has expressed its support for a multi-specialist evaluation.

Table 3 shows the summary of the drafted recommendations.

The panel of experts reiterates the importance of using povidone iodine for peri-operative prophylaxis in elective intra- and extraocular surgeries, as unequivocally underlined by the available literature. It is also recalled that in clinical practice it is common to administer topical antibiotic therapy, through eye drops, in the peri- and postoperative period, but the indication for this type of approach seems limited. In addition, it is common in the postoperative period to use topical medications such as steroids and nonsteroidal anti-inflammatory drugs (NSAIDs), alone or in combination, for anti-inflammatory purposes in order to accelerate healing and reduce patient discomfort after surgery [103,104,105]. For possible indication for postoperative prophylaxis with eye drops and how to administer them, the patient should be referred to the ophthalmic specialist, with close follow-up.

In our manuscript, we did not include a specific pharmacoeconomic section. However, when antibiotics are recommended, we always suggest narrow-spectrum antibiotics with low cost. This means that our consensus document can be useful also in limited-resources countries.

In this work, the formulation of scenarios is aimed at guiding the healthcare professional in practice, in order to ensure a better and standardized management of neonatal and pediatric patients, together with an easy consultation. The strengths of the work are an up-to-date review of the literature, the use of a rigorous method of analysis (RAND/UCLA), and the involvement of several representatives of the most important Italian scientific societies. The potential limitation of the work is the scarcity of data in the literature and the main reference to adulthood, partly overcome by the involvement of numerous and selected experts. Another limitation is that this was an opinion-based survey. On the other hand, the lack of pediatric studies on the selected topics did not permit the use of GRADE methodology, and the complexity of the topics required an online face-to-face meeting with all participants.

## 5. Conclusions

This work has been made possible by the multidisciplinary contribution of experts belonging to the most important Italian scientific societies and represents, in our opinion, the most complete and up-to-date collection of recommendations regarding the clinical actions in the peri-operative environment in eye surgery. The application of uniform and shared protocols aims to improve surgical practice, through the standardization of procedures, with a consequent reduction of SSIs, also limiting the phenomenon of antimicrobial resistance. In order to overcome barriers or hurdles, a strong educational activity associated with tools such as audit and feedback as a moment of “self-analysis” of a health organization, focus groups that give space for discussion, and the support of the political decision-maker are key elements for the success of the implementations of these recommendations at a local level.

## Figures and Tables

**Table 1:** Major ocular surgeries in neonatal and pediatric age.

Intraocular Surgery Lens surgery (infantile cataract) Trabecular meshwork surgery (infantile glaucoma) Vitreoretinal surgery [(retinopathy of the premature; hereditary vitreoretino pathies; retinal detachment (cerclage and/or scleral lead, vitrectomy, gas retinopexy)] Iris surgery: iridectomy or iridotomy (for glaucoma) Corneal surgery with full-thickness cutting
Extraocular Surgery Lacrimal tract surgery Eyelid surgery (ptosis; blepharitis; chalazion; stye) Conjunctiva surgery (foreign body removal; conjunctiva repair; lesion removal) Corneal surgery, without full-thickness involvement Eye muscle surgery: strabismus correction Paralytic strabismus (innervation deficit of the eye muscles caused by congenital malformations, traumatic or inflammatory injuries or outcomes of surgery on the eyeball) Non-paralytic strabismus (due to a malfunction of the nerve centers responsible for coordinating eye movements)
Surgery for Ocular Trauma
Corneal Transplant Surgery
Surgery for Tumor Removal

**Table 2:** Main neoplasms with indications of the recommended therapy.

Neoplasia	Surgery	Other Therapy *
Retinoblastoma	yes	yes
Ocular superficial squamous neoplasm	yes	yes
Melanoma of the conjunctiva	yes	yes
Conjunctival lymphoma	yes	yes
Nevus of conjunctiva	yes	no
Melanoma of the uvea	yes	yes
Lymphoma of the uvea	no	yes
Vitreous and retinal lymphoma	no	yes
Uveal metastases	no	yes

* Other therapies include chemotherapy, hormone therapy, intra-lesional injection of monoclonal antibodies, brachytherapy, immunotherapy, and radiotherapy.

**Table 3:** Recommendations regarding peri-operative antibiotic prophylaxis in neonatal and pediatric patients undergoing eye surgery.

Eye Surgery	Antibiotic Prophylaxis	Molecule
Clean intraocular eye surgery	Yes	Intrachamber cefuroxime 1 mg
Clean extraocular eye surgery	No	-
Intervention for ocular-penetrating trauma	Yes	Intraocular vancomycin and ceftazidime. The administration of vancomycin 15 mg/kg (max 1 g) IV in combination with ceftazidime 50 mg/kg (max 2 g) IV is recommended in cases in which the risk of infection is increased (i.e, age <1 year, poor initial visual acuity, posterior involvement, extensive injury, vitreous hemorrhage, retinal detachment, and endophthalmitis)
Ocular neoplasm surgery	No	-
Ocular surface transplantations	Yes	Multi-specialist evaluation
Corneal grafts	Yes	Multi-specialist evaluation

## Data Availability

All the data are included in the manuscript.
